# Prognostic value of LINC02560 in colorectal cancer correlates with tumor microenvironment immunity

**DOI:** 10.7150/jca.64940

**Published:** 2021-11-04

**Authors:** Chunying Luo, Fahui Liu, Weichao Su, Puze Long, Jiadong Liang, Wanyun Hou, Haifeng Jiang, Xidai Long, Guoqiang Su

**Affiliations:** 1 Department of Cell Biology, Medical College of Guangxi University, Nanning 530004, Guangxi, PR China.; 2 Department of Pathology, Affiliated Hospital of Youjiang Medical University for Nationalities, Baise 533000, Guangxi, PR China.; 3 Department of Gastrointestinal Surgery III, Xiamen Cancer Hospital, First Affiliated Hospital of Xiamen University, 55 Zhenhai Road, Xiamen 361003, China.

**Keywords:** Colorectal cancer, LINC0256, Prognosis, Tumor immune microenvironment

## Abstract

**Background:** LINC02560 is a new 477 bp long non-coding RNA located in 19q13.43. However, the expression of LINC02560 in colorectal cancer (CRC) has not been reported, and its correlation with tumor development and function is still unclear.

**Methods:** The expression of LINC02560 in CRC was first analyzed in the cancer genome atlas (TCGA) combined with The Genotype-Tissue Expression(GTEx) databases and then validated by clinical CRC samples and cell lines. The association between LINC02560 expression and clinicopathologic variables was analyzed by the Wilcoxon Rank SUM test. Cox regression analysis and Kaplan-Meier plots were used to assess the prognostic value of LINC02560 in CRC. The correlation between the expression level of LINC02560 and the 24 immune cells in tumor microenvironment (TME) was analyzed by single sample gene set enrichment analysis (ssGSEA). Gene set enrichment analysis (GSEA) was conducted to detect potential biological processes associated with LINC02560 in CRC.

**Results:** LINC02560 was significantly up-regulated in CRC in comparison to normal samples. There are significant differences in the expression of LINC02560 in different subgroups of N stage, M stage, carcinoembryonic antigen (CEA) level, residual tumor, TP53 status and pathological stage. The high LINC02560 expression indicated poor overall survival (OS) and progress free interval (PFI) in patients with CRC. Moreover, the multivariate Cox analysis demonstrated that the expression of LINC02560 was an independent prognosis-predicting factor for OS in CRC patients. GSEA indicated that high expression of LINC02560 was involved in MAPK, Wnt, and PPAR signaling pathways and participated in humoral immune processes. We also identified that LINC02560 expression had a negative correlation with 4 kinds of immune cells.

**Conclusions:** In summary, our research results indicate that LINC02560 may be a potential prognostic biomarker. It is involved in the occurrence and development of CRC and may affect the prognosis of CRC patients by regulating immune cells in the TME.

## Introduction

CRC is still predominately a cancer of older individuals. CRC is one of the most common malignant tumors. In 2018, morbidity and mortality in the United States ranked 4^th^ and 2^nd^ in the cancer spectrum 1. However, colon cancer is becoming more prevalent in younger individuals 2. The incidence and mortality of CRC in China rank fifth. However, the death to new cases (50.8%) ratio is significantly higher than that of the United States (36.6%).

Genetic factors are one of the causes of colon cancer. Approximately 30% of patients with CRC have a family history of CRC. Many factors promote the occurrence and development of colon cancer, mainly including gene mutation, inflammatory response, immunity, non-coding RNA regulation disorder and the activation of some oncoproteins. At present, the primary treatment for CRC is a combination of surgical resection, chemoradiotherapy and immunotherapy. Monoclonal antibodies against epidermal growth factor receptor (EGFR) are used to treat CRC that has metastasized. The 8th edition of the American Joint Committee on Cancer (AJCC) cancer staging system establishes a “non-anatomical” prognostic risk and curative effect prediction evaluation system based on molecular testing results. Microsatellite instability and BRAF gene status were used as prognostic factors, and BRAF, KRAS and NRAS gene mutation status were used as curative effect predictors 3. The above vital genes all show the clinical application value of biomolecular markers.

As we all know, tumors have apparent heterogeneity. Tumors of the same type or the same stage may significantly differ in their biological behavior and prognosis. Genomic changes drive the occurrence and development of cancer, and the differences in gene changes in tumor cells indicate different prognostics, treatment methods and therapeutic effects. Many factors promote the occurrence and development of colon cancer, mainly including gene mutation, inflammatory response, immunity, non-coding RNA regulation disorder and the activation of some oncoproteins. Nevertheless, the level of evidence for each biomolecular marker is between I-II 3. The combined evaluation of multiple markers is more effective and clinically valuable. Therefore, it is of great scientific and clinical significance to find and study more specific biomolecular markers for CRC.

With the in-depth study of non-coding RNA, it has been found that it exhibits essential biological functions due to its extensive and important gene expression regulation function. Long-chain non-coding RNA is the most versatile non-coding RNA. It is involved in gene transcription, translation, post-transcriptional and post-translational level regulation and epigenetic regulation. It may play an essential role in the occurrence and development of tumors and may become new molecular markers for tumor prognosis, diagnosis and efficacy judgment.

LINC02560 is a new long non-coding RNA located in 19q13.43 with 477 bp. It has been reported that bronchial epithelial cells are expressed in Pseudomonas aeruginosa infection 4. Nevertheless, the expression of LINC02560 in tumor tissues has not been reported, and the correlation between its expression and tumor development and its function is still unclear. Our research intends to have an initial exploration of the above questions. In this study, bioinformatics analysis, clinical samples and tumor cell lines were used to analyze the expression difference, function and clinical significance of LINC02560 in CRC tumors from multiple perspectives.

## Materials and Methods

### RNA-sequencing data and bioinformatics analysis

Gene expression data (HTSeq-FPKM) with clinical information of CRC samples were collected from TCGA (https://cancergenome.nih.gov/) in January 2021. At the same time, cases with OS less than 30 days were excluded. Finally, 619 primary tumors with both clinical and gene expression data were used for further analysis. Level 3 HTSeq-FPKM was then transformed into TPM for the following research. In addition, we download the GTEx RNA-Seq data (TPM), which was processed using the TOIL pipeline 5. We acquired pan‐cancer data of gene expression from the UCSC XENA (https://xenabrowser.net/datapages/) for pan-cancer analysis.

### Clinical specimens and cell lines

A total of 10 patients with CRC who underwent surgical resection in the Affiliated Hospital of Youjiang Medical University for Nationalities were collected. Inclusion criteria were the following: All enrolled patients did not receive any preoperative therapies before surgery and sample collection and were characterized according to the 8th edition of the AJCC staging manual. CRC confirmed by written medical report. Patients with metastatic CRC and those with past history of other cancer were excluded. This study was reviewed and approved by the Ethics Committee of the affiliated Hospital of Youjiang Medical University for Nationalities and all patients agreed with written informed consents (YYFY-LL-2021-16). All samples were well preserved at -80°. This study was approved by the Academic Committee of the Affiliated Hospital of Youjiang Medical University for Nationalities and conducted according to the Helsinki Declaration's requirements. In addition, SW-480 (ATA-CL1052), SW1116 (ATA-CL042) and HCT-116 (CL0125) were purchased from PuJian Cell Center (Wuhan, China) and FengHui Cell Center (Beijing, China) respectively, human normal colon epithelial cells NCM-460 (ATA-CL1041) were purchased from PuJian Cell Center, (Wuhan, China). All cells were cultured at 37 °C and in a 5% CO^2^ constant humidity environment.

### The expression and localization of LINC02560 in CRC

RT-qPCR was used to analyze the difference in LINC02560 expression between tumor samples and normal tissues. Similarly, RT-qPCR is also used to analyze the expression differences of LINC02560 between different cell lines. Follow the instructions of the Trizol reagent to extract total RNA. Moreover, its concentration and purity were quantified by a spectrophotometer. Use reverse transcription kit to synthesize cDNA, using cDNA as template, add Real-SYBR Mixture, upstream and downstream primers and RNase-free water to configure the reaction system, using 2^-ΔΔ^ CT method to calculate the relative expression of gene RNA. The LINC02560 primer sequence is as follows: sense sequence: 5'-TCGCTCCTAGGTGCTTTTGT-3'; antisense sequence: 5'-AAAACAGAATCAAAGGCCGTGC-3'. The β-Actin primer sequence is as follows: sense sequence: 5'-CTCTTCCAGCCTTCCTTCCT-3' antisense sequence: 5'-AGCACTGTGTTGGCGTACAG-3'. The localization of LINC02560 in CRC was detected by RNA *in situ* hybridization. The probe sequence of LINC02560RNA was designed as follows: 5-Cy3-AAGAGGAGAUUGGCUGAGGCUGGGCAAGGGU-3.

### Bioinformatics analysis

The 619 patients with CRC were cut off by the high expression group (n=309) and low expression group (n=310) according to the median expression level of LINC02560. In our research, GSEA carried out by R package “cluster profile”(3.14.3) was used to elucidate the significant functions and pathways difference between high- and low- LINC02560 groups. Gene set permutations were performed 1000 times for each analysis. A function or pathway term with P-value<0.05, FDR q*-*values <0.25, and Normalized enrichment score (|NES|>1) was considered statistically significant enrichment. The immune cells infiltration analysis of CRC was performed by the ssGSEA method to explore the correlation between the LINC02560 expression and the infiltration levels of immune cells. Spearman's correlation analyzed the correlation between LINC02560 and 24 types of immune cells. The Wilcoxon rank-sum test was used to clarify the association of infiltration of immune cells in the different expression groups of LINC02560.

### Statistical analysis

All statistical analyses were analyzed using R (v4.0.2). The different expression of LINC02560 between tumor and normal tissues was calculated using the Wilcoxon rank-sum test and Wilcoxon signed-rank test. Kruskal-Wails test and Wilcoxon signed-rank test were used to assess the relationships between LINC02560 expression and clinic-pathological features. Prognostic factors were evaluated by using the Kaplan-Meier method and uni- and multivariate analyses. In all analyses, P*-*values < 0.05 were considered statistically significant.

## Results

### LINC02560 expression status in pan-cancer and CRC

**Figure 1:**
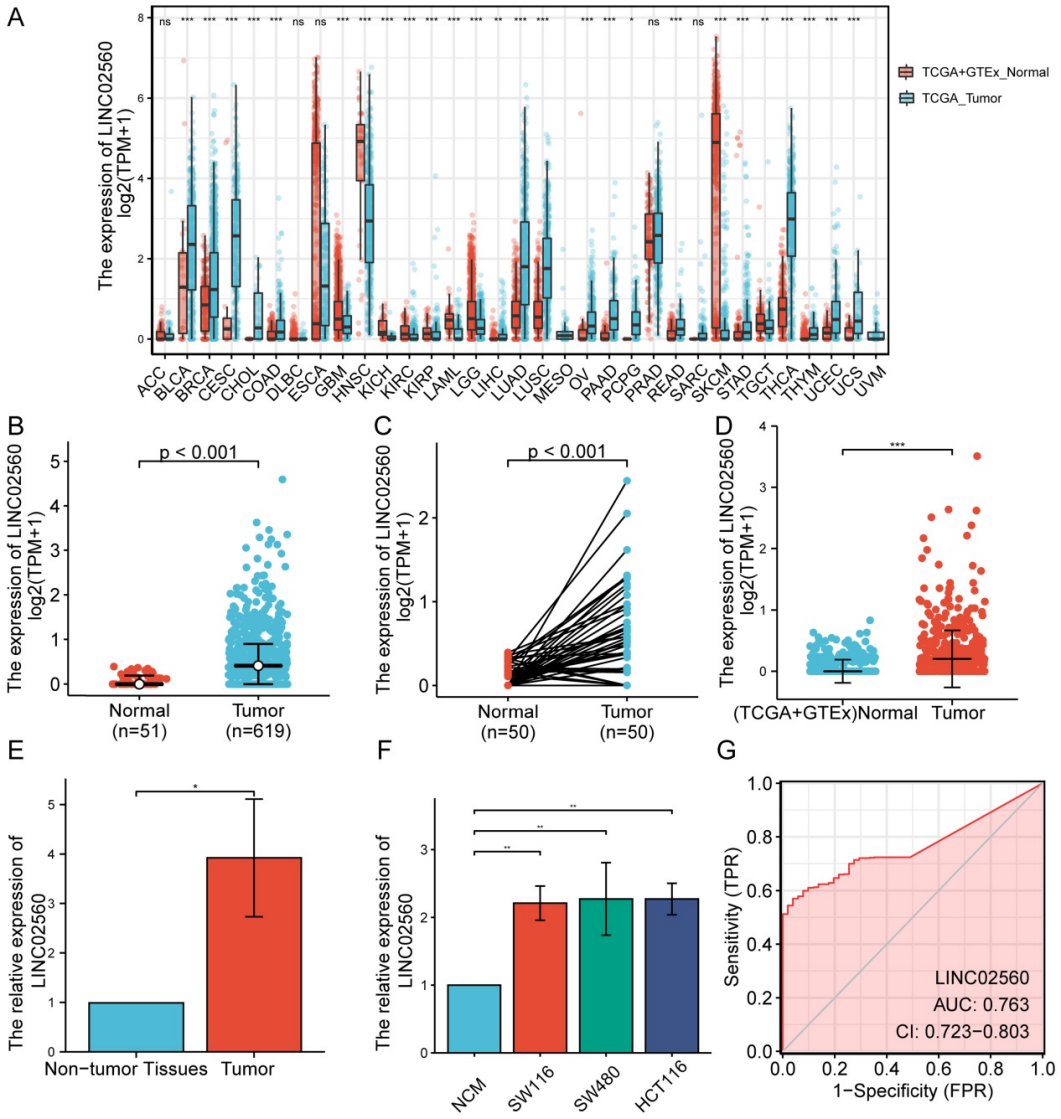
** Expression of LINC02560 in tumors and evaluation of its diagnostic efficacy. A:** Pan-cancer analysis of LINC02560 expression. **B:** The expression of LINC02560 was higher in CRC than in normal tissues. **C:** The differential expression of LINC02560 in 50 paired CRC tumor and adjacent normal tissues in TCGA. **D:** The differential expression levels of LINC02560 from TCGA tumor tissue versus combined normal TCGA and GTEx data. E: Relative expression of LINC02560 in between CRC tissues and adjacent non-tumor tissues. **F:** Relative expression of LINC02560 in different cell lines. **G:** The diagnostic efficacy of LINC02560 in CRC.

The results of the pan-cancer analysis showed that LINC02560 in bladder urothelial carcinoma, invasive breast carcinoma, cholangiocarcinoma, colon adenocarcinoma, glioblastoma multiforme, head and neck squamous cell carcinoma, kidney chromophobe, kidney renal clear cell carcinoma, kidney renal papillary cell carcinoma, acute myeloid leukaemia, brain lower grade glioma, hepatocellular carcinoma, lung adenocarcinoma, lung squamous cell carcinoma, ovarian serous cystadenocarcinoma, pancreatic adenocarcinoma, pheochromocytoma and paraganglioma, rectum adenocarcinoma, skin cutaneous melanoma, stomach adenocarcinoma, testicular germ cell tumors, thyroid carcinoma, thymoma, uterine corpus endometrial carcinoma, and uterine carcinosarcoma were significantly differentially expressed (P<0.05) (Fig. 1A). The expression level of LINC02560 in the tumor was significantly higher than that in normal tissues (Fig. 1B). Moreover, Wilcoxon signed-rank test was used to compare the expression of LINC02560 in 50 CRC samples and the corresponding paired para-cancer samples in TCGA. The results showed that LINC02560 was highly expressed in paired CRC samples (P< 0.001) (Fig. 1C). In addition, we compare the expression of LINC02560 in normal samples of GTEx combined TCGA and CRC samples of TCGA, and it was found that LINC02560 was highly expressed in CRC samples (P<0.001) (Fig. 1D). Also, the relative expression of LINC02560 in 10 pairs of clinical samples and different cell lines was detected by qRT-PCR. The results showed that the relative expression of LINC02560 in CRC tissues was higher than that in adjacent normal tissues (Fig. 1E). The relative expression of LINC02560 in tumor cell lines was significantly higher than that in normal colon epithelial cells (Fig. 1F). ROC analysis was applied to validate the potential diagnostic of LINC02560, and the area under the ROC curve was calculated for LINC02560. The area under the ROC curve is 0.763, suggesting that LINC02560 may be a potential diagnostic marker (Fig. 1G). The result RNA *in situ* hybridization shows that LINC02560 is localized and expressed in both nucleus and cytoplasm in CRC (S1).

### The clinical characteristics of CRC patients in TCGA

The characteristics of 619 CRC patients from TCGA , including T stage, N stage, M stage, CEA level, History of colon polyps, Colon polyps present, Neoplasm type, Residual tumor, Lymphatic invasion, Pathologic stage, Gender, Primary therapy outcome, Age, were collected and shown in Table 1.

### Association between LINC02560 expression and clinicopathology variables

**Figure 2:**
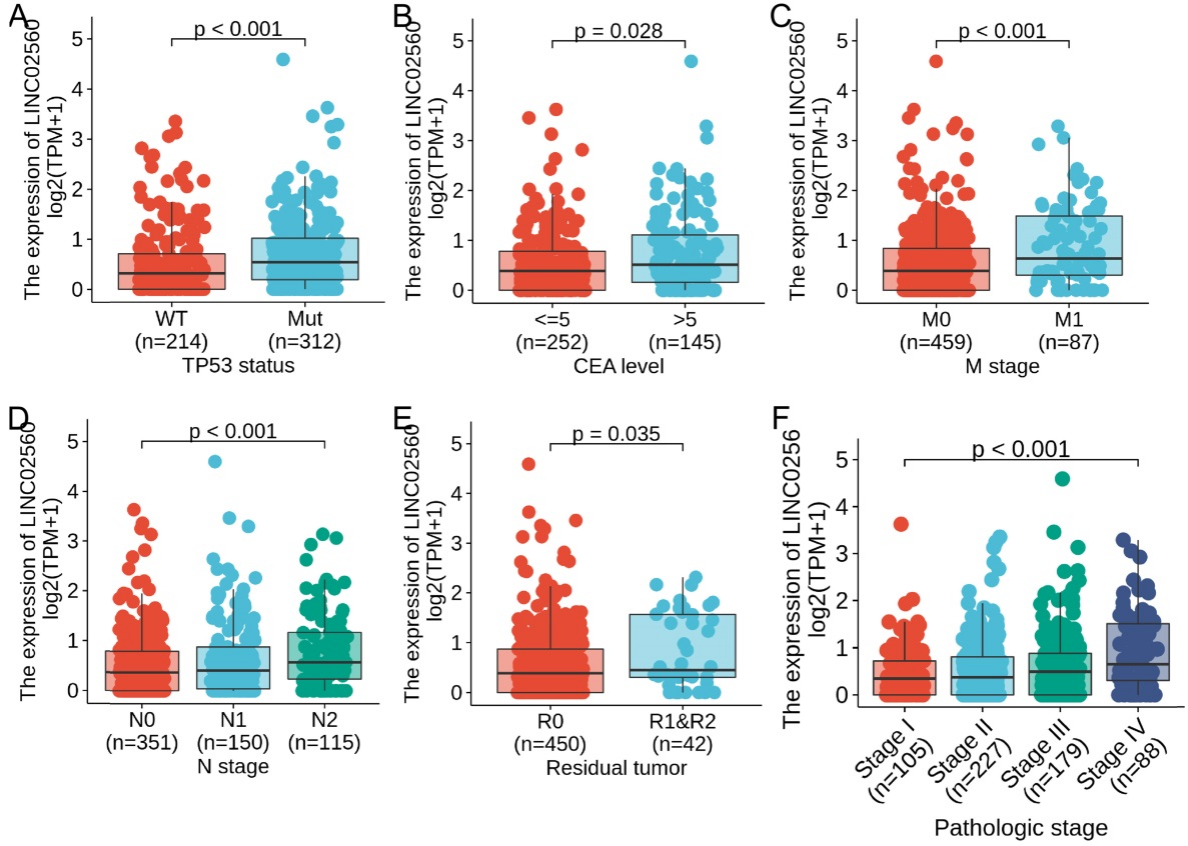
Expression differences of LINC02560 in different subgroups of CRC.

In order to further understand the clinical significance of LINC02560 in CRC, we compared the clinicopathological relationship between LINC02560 expression and different subgroups. The results showed that there are significant differences in the expression of LINC02560 in different subgroups of N stage (P<0.001), M stage (P<0.001), CEA level (P=0.028), residual tumor (P=0.035), TP53 status (P<0.001) and pathological stage (P<0.001) (Fig. 2A-F). These results indicated that CRC with increased LINC02560 expression could progress to an advanced stage and metastasis.

### LINC02560 was independent prognostic factors for OS in CRC

A Kaplan-Meier diagram drawn by the “survminer” package was used to evaluate the prognostic value of LINC02560 in CRC. Results showed that high expression of LINC02560 was associated with worse OS (HR=1.42 (1.00-2.02), P=0.048) and progress free interval (HR=1.42 (1.04-1.95), P=0.029). In the Cox regression model, variables with p<0.05 in the univariate Cox regression will be included in the further multivariate Cox regression. The variables that meet this threshold are T stage (P=0.006), N stage (P<0.001), M stage (P<0.001), Pathologic stage (P<0.001), CEA level (P<0.001), Age (P<0.001), Residual tumor (P<0.001) and LINC02560 (P=0.026). Multivariate Cox regression found that Pathologic stage (P= 0.012), Age (P=0.002), and LINC02560 (P=0.041) was independent prognostic factors for OS (p<0.05) (Table 2).

### Related signaling pathways and function of LINC02560 based on GSEA

GSEA was used to identify signaling pathways correlated with/involved in CRC between high and low expression of LINC02560 expression data sets, and demonstrated significant differences (FDR<0.05, adjusted P-value<0.05)in enrichment of MSigDB Collection (C2.all.v6.2.symbols.gmt). A total of 9 functions and/or pathways, including immune response regulating cell surface receptor signaling pathway, humoral immune response, antigen receptor-mediated signaling pathway, were significantly enriched in low expression of LINC02560. Furthermore, negative regulation of epithelial to mesenchymal transition, the PPAR signaling pathway, WNT signaling pathway, basal cell carcinoma, MAPK signaling pathway and regulation of actin cytoskeleton were significantly enriched in high expression of LINC02560. The results indicated the potential role of LINC02560 in the development of CRC.

### The correlation between LINC02560 expression and immune cells infiltration

**Figure 4:**
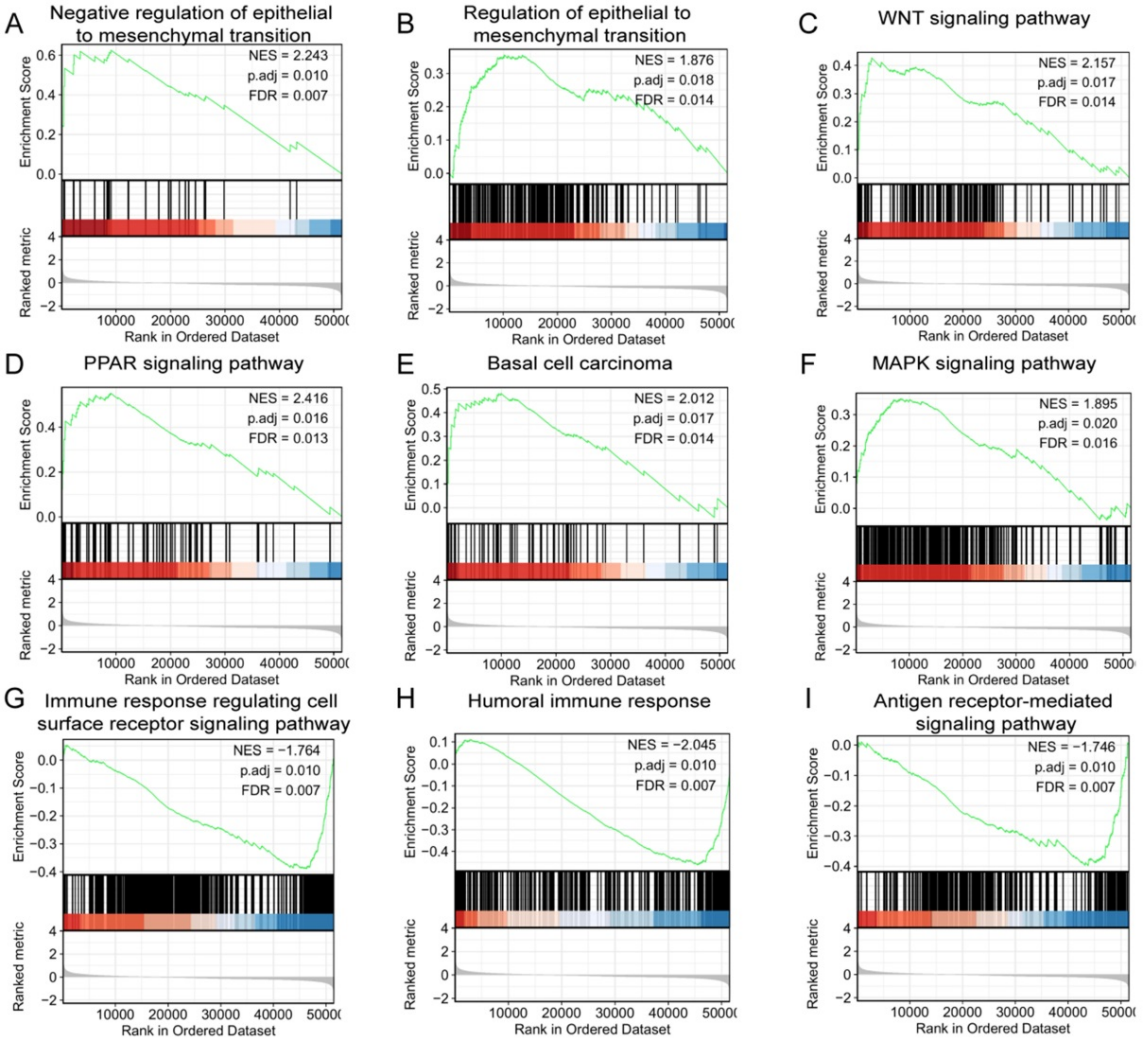
LINC02560 relative signaling pathways and functions.

The correlation between the expression level of LINC02560 and the immune cells infiltration in TME of CRC patients was quantified by ssGSEA and analyzed by Spearman correlation. The lollipop chart showed a correlation between LINC02560 and levels of 24 immune cell infiltrates, and it turned out that the expression level of LINC02560 was significantly correlated with multiple immunocytes (Fig. 4A). Specifically, Cytotoxic cells (r=-0.115, P=0.004), CD8 T cells (r=-0.095, P=0.018), T helper cells (r=-0.093, P=0.020), DCs (r=-0.092, P=0.022), Th1 cells (r=-0.091, P=0.023), Th2 cells (r=-0.233, P<0.001), aDCs (activated DCs) (r=-0.175, P<0.001), B cells (r=-0.086, P=0.032)and T cells (r=-0.116, P<0.05) are correlated with the expression of LINC02560, but there is no correlation between LINC02560 and other types of immune cells in CRC. Also, 4 types of immune cells (Th2 cells, T cells, aDCs, cytotoxic cells) showed different expressions in different groups of LINC02560 (Fig. 4B-E). We further analyzed the correlation between the 4 types of immune cells and the expression of LINC02560. The expression of LINC02560 was significantly negatively correlated with the infiltration of 4 types of immune cells (Fig. 4F-I). It shows that the different expression of LINC02560 in CRC affects the tumor immune microenvironment. Tumors with high LINC02560 expression have reduced cellular immunity and antigen presentation ability in the microenvironment.

## Discussion

According to the CRC staging in the 8^th^ edition of AJCC, serum carcinoembryonic antigen (CEA) levels, RAS, BRAF gene mutations, microsatellite instability, and tumor regression scores are recommended as molecular markers for predicting the prognosis risk and curative effect of CRC 3. Studies have found that high preoperative CEA serum levels are associated with poor prognosis of patients with CRC 6. Postoperative serum CEA levels are also an independent prognostic factor in CRC. Both KRAS and NRAS gene mutations suggest that stage III to IV CRC has a poor prognosis and an inadequate response to anti-EGFR targeted therapy. BRAF gene mutations predict the poor efficacy of anti-EGFR targeted drugs. MSI is often used for colon cancer prognosis judgment and medication guidance. MSI-H indicates a good prognosis and a poor effect on 5-FU chemotherapy 7.

At present, there has been researching on long non-coding RNA in CRC, but little is known about the expression, specific function and clinical value of known long non-coding RNA. New long non-coding RNA and its new functions are constantly being discovered and recognized. It is necessary and meaningful to explore further the relationship and regulations of the occurrence, development, and evolution of long noncoding RNA and CRC. Most lncRNAs are located in the nucleus and mainly play a regulatory role at the molecular level 8. They can regulate the proliferation, invasion, apoptosis, metastasis and other biological processes of CRC, and even can be used as biomarkers for prognosis, diagnosis and curative effect prediction of CRC 9, 10. Current studies have shown that the overexpression of lncRNA-p21 can inhibit the migration and invasion of straight colon cancer cells and can be used as an effective tumor inhibitor, which is expected to become a therapeutic target 11. Another study found that in CRC tissues, high expression of lncRNA-TUG1 is closely associated with rapid disease progression and mediates 5-fluorouracil resistance by regulating miR-197-3p in CRC 12. In conclusion, the above studies indicate that lncRNA plays a vital role in CRC. At present, we found that LINC02560 was overexpressed in CRC through database mining and analysis. Therefore, in this study, we identified the expression of LINC02560 in the database, CRC cell lines and clinical samples. Furthermore, by using the database to verify further the clinical significance and function of the molecule in CRC.

In this study, we first analyzed the expression status of LINC02560 in different types of human cancers. The results showed that LINC02560 was significantly highly expressed in 24 cancer tissues, suggesting that it may act as an oncogene to promote tumors. In addition, we discussed the clinical significance and application value of LINC02560 by analyzing CRC samples from the TCGA database. The results showed that LINC02560 was significantly overexpressed in CRC samples, and CRC patients with high LINC02560 expression had poorer OS. At the same time, we conducted verification tests on the expression of LINC02560 in clinical CRC samples and cell lines, which confirmed the conclusion that LINC02560 was significantly overexpressed in CRC. In addition, LINC02560 is also significantly overexpressed in many more advanced patients, which indicates that higher expression levels of LINC02560 have a positive effect on the progression of CRC. Finally, through the analysis of GSEA and ssGSEA, it is found that LINC02560 may be related to the infiltration of immune cells in TME and is mainly involved in immune functions and pathways.

First, we compared the expression of LINC02560 in normal samples and CRC samples of TCGA. It was found that LINC02560 was highly expressed in CRC samples, and the results were statistically significant (P<0.001). In addition, the results of the high expression of LINC02560 in CRC tumor tissues have been verified in our own 10 fresh CRC tumors and para-cancerous samples. This confirms the role of LINC02560 as an oncogene in CRC. We evaluated the diagnostic value of LINC02560 as an oncogene for CRC through ROC analysis. The area under the ROC curve is 0.763. These findings suggest that LINC02560 may be a potential diagnostic marker. We will further try to detect the level of LINC02560 in the serum of CRC patients and verify and evaluate the clinical value of LINC02560 as a diagnostic marker in our cases.

In order to further understand the clinical significance of LINC02560 in CRC, the relationship between LINC02560 and clinicopathological features. On the one hand, there are significant differences between LINC02560 and CEA at different levels, indicating the feasibility of LINC02560 as a diagnostic marker, which is consistent with the results of our previous analysis. On the other hand, LINC02560 has significant differences in N stage, M stage, Residual tumor, TP53 status, and pathologic stage in different subgroups. At the same time, we also noticed that LINC02560 is always highly expressed in more malignant tissues. This indicates that LINC02560 is involved in tumor progression and evolution, promoting tumor proliferation and lymph node metastasis. However, its exact influence mechanism and function urgently need to be further studied.

Given the abnormally high expression of LINC02560 in CRC with higher levels of malignancy, the prognosis for these patients is generally poor. We further analyzed and explored the prognostic role of LINC02560 in CRC. First, we evaluated the prognostic value of LINC02560 in CRC and found that the high expression group of LINC02560 had a worse OS than the low expression group (HR=1.49 (1.05-2.13), P=0.026). Cox regression analysis showed that LINC02560, CEA, pathological grade and age were independent prognostic factors in CRC patients. The expression of LINC02560 indicates the possibility of LINC02560 as a prognostic indicator of CRC. In the next step, we will further verify its prognostic value in our own CRC clinical samples and cases and actively evaluate it as a prognostic judgment of CRC through evidence-based medical research.

GSEA analysis showed that the enriched functions mainly involved cell recognition, site recognition and signal transduction, humoral immune response, etc. The signaling pathways enriched and related to the high expression of LINC02560 mainly include the MAPK signaling pathway, Wnt signaling pathway, PPAR signaling pathway, regulation of actin cytoskeleton and Basal cell carcinoma. The MAPK signaling pathway is a vital transmission pathway of signals from the cell surface to the nucleus. It is a group of serine-threonine protein kinases activated by different extracellular stimuli, such as cytokines, neurotransmitters, hormones, cell stress and cell adhesion. It regulates many critical cellular physiological and pathological processes such as cell growth, differentiation, environmental stress adaptation, and inflammatory response. Studies have reported that BRAF/KRAS induces the activation of the MAPK/ERK signaling pathway, triggering colon cancer 13. The PPAR pathway is a member of the nuclear receptor transcription factor superfamily that regulates the expression of target genes 14. In recent years, it has been found that PPARs are closely related to energy (lipid, sugar) metabolism, cell differentiation, proliferation, apoptosis, and inflammatory reactions. It has been found that PPARγ is highly expressed in normal colon cells, highly differentiated and poorly differentiated CRC cells, and promotes the proliferation and differentiation of tumor cells 15, 16. Our results show that in the group with high expression of LINC02560, the PPAR signaling pathway is activated, which further proves that LINC02560 is closely related to CRC and provides a reliable direction for further development mechanism research.

The Wnt signal transduction pathway is a set of multi-downstream signal transduction pathways stimulated by binding to the ligand-protein Wnt and the membrane protein receptor. Activation of the Wnt pathway can promote tumor proliferation and the EMT process and participate in development and organ formation. The classical Wnt signaling pathway plays a vital role in the occurrence and development of colon cancer 17, 18. Frequent mutations of Wnt signaling components are closely related to the occurrence of colon cancer, and the abnormally activated Wnt/β-catenin signaling pathway promotes the occurrence of colon cancer. More than 80% of colon cancer tumors are found to have a lack of APC function, and about 5% of colon cancer tumors carry mutation-activated β-catenin 17, 19.

We analyzed the association between LINC02560 expression level and 24 kinds of immune cell infiltration in tumor tissue and found that LINC02560 expression level was negatively correlated with Th2 cells (r=-0.233, P<0.001), ADCs (Activated DCs) (r=-0.175, P<0.001), Cytotoxic cells (r=-0.115, P=0.004), CD8 T cells (r=-0.095, P=0.018). The results suggested that Th2 cells, ADCs and Cytotoxic cells infiltration in CRC patients with LINC02560 high expression was reduced, and the immune microenvironment showed immunosuppression. LINC02560 is expected to be a target of immunotherapy and provide a direction for further research.

It has been shown that the immune system plays an important role in the development of colon cancer 20. A large subpopulation of T cells typically infiltrates carcinoma *in situ* 21-23. Patients with colon cancer with abundant infiltration of CD8-positive T cells have a decreased postoperative recurrence rate and a significantly increased survival rate 22-24. In particular, the high-density cytotoxic memory T cells in the primary neoplasm can reduce the postoperative recurrence rate and metastasis rate of fully clothed colon cancer 25, 26. CD4 positive T cells expressing FOXP3 as regulatory T cells (Treg) block the immune response to cancer cells 27, 28. Rich infiltration of Treg cells in tumors is associated with a poor prognosis for various cancers 21, 29, 30. However, in colon cancer, the role of Treg cells is controversial. Zhu et al. 31 found that FOXP3-positive T cells promoted the development of colon cancer by inhibiting the immune response of colon cancer. Other studies have shown that infiltration of FOXP3-positive T cells predicts better prognostic outcomes 21, 30, 32-34. Based on previous studies, Saito et al. further found that in colon cancer tissue, patients infiltrating many T cells with low expression of Foxp3 had a better prognosis than patients infiltrating a large number of T cells with high expression of Foxp3 35. Therefore, FOXP3 expression in different T cell subsets determines a good prognosis in patients with colon cancer.

This study studied the expression of LINC02560 in CRC and its clinical significance by TCGA database mining and clinical sample verification. The results showed that LINC02560, as a new oncogene, promotes the tumor progression of CRC and is an independent prognostic biomarker for poor prognosis of CRC. Its effect may be related to cell and site recognition and humoral immune response. Its mechanism may be that it promotes tumorigenesis through MAPK signaling pathways and Wnt signaling pathways. In this study, clinical specimens were used to verify the expression of LINC02560 in CRC tissues. We will further increase the number of clinical specimens to verify the expression, function, clinical significance and mechanism of action and further evaluate the value of LINC02560 as a prognostic and diagnostic evaluation indicator of CRC. Meanwhile, we will further build a prognostic risk model for CRC to help clinicians judge the prognosis of patients and evaluate the biological behavior of tumors, to provide the best “individualized” treatment plan for patients.

## Figures and Tables

**Figure 3:**
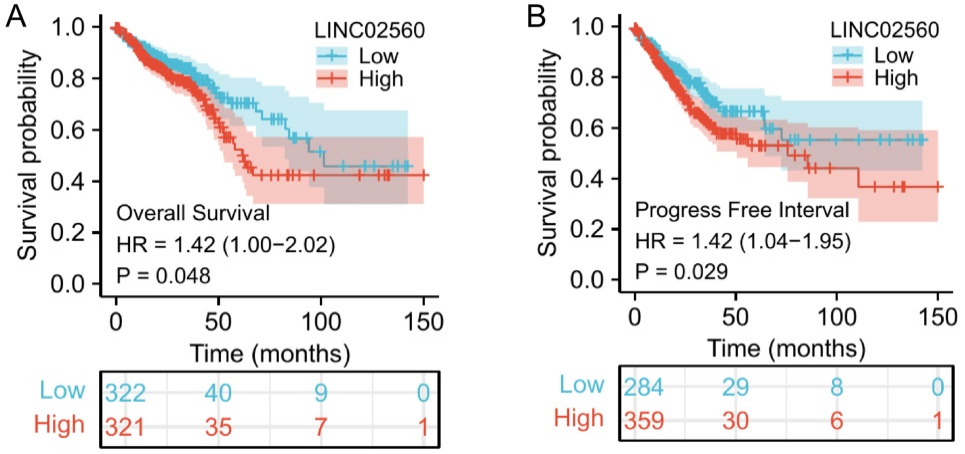
Effect of LINC02560 expression on overall survival and progress free interval in CRC.

**Figure 5:**
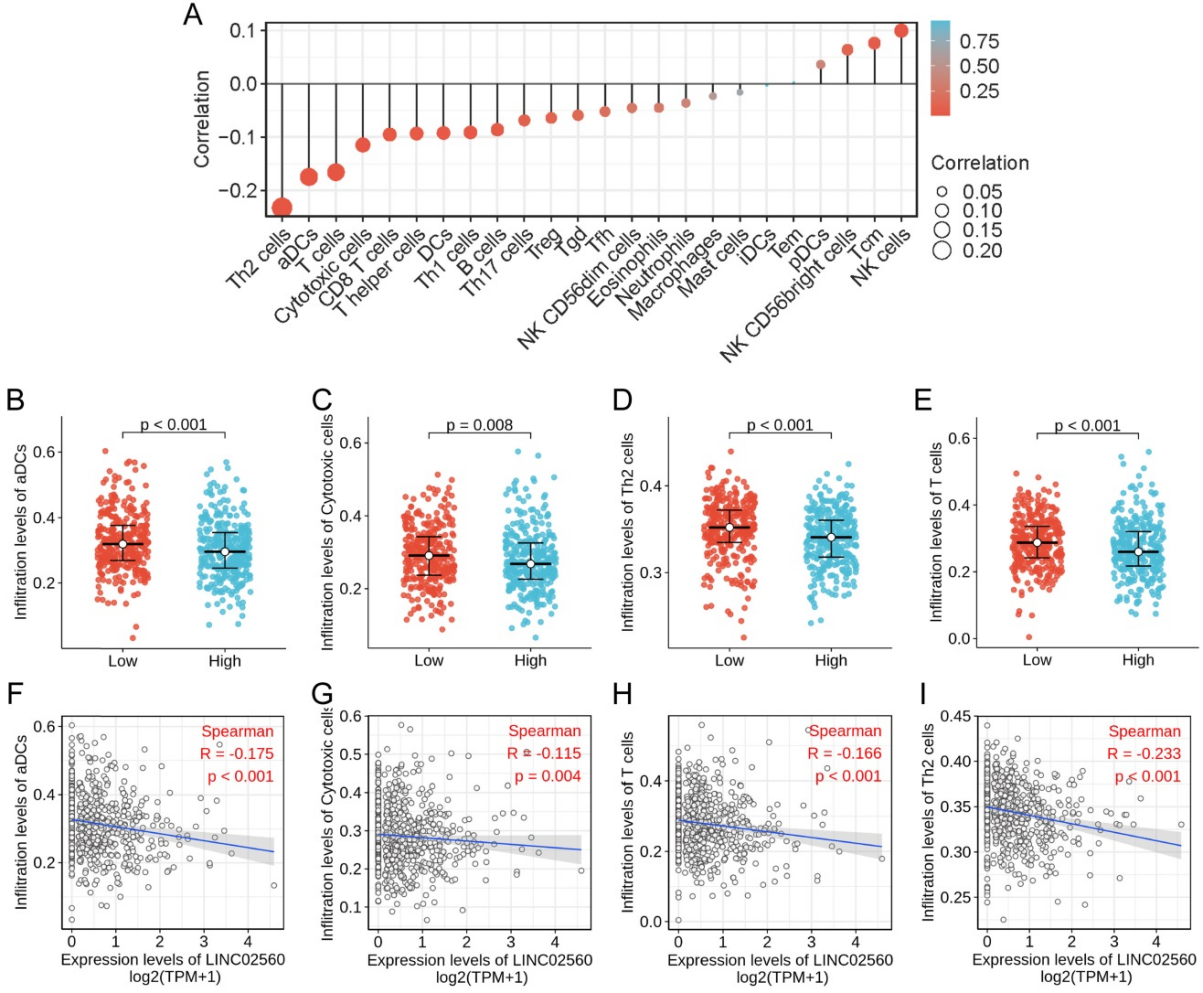
** The relationship between LINC02560 expression and immune cells infiltration. A:** Lollipop diagram showing a correlation between LINC02560 and levels of 24 immune cell infiltrates. **B-E:** 4 kinds of immune cells are differentially expressed in different groups of LINC02560 (P< 0.05). **F-I:** Scatter plot showed the correlation between the infiltration of 4 types of immune cells and the LINC02560 expression (P < 0.05).

**Table 1:** Detailed clinical information for all CRC patients from TCGA

Characteristic	levels	Overall
T stage, n (%)	T1	20 (3.1%)
	T2	111 (17.3%)
	T3	436 (68%)
	T4	74 (11.5%)
N stage, n (%)	N0	368 (57.5%)
	N1	153 (23.9%)
	N2	119 (18.6%)
M stage, n (%)	M0	475 (84.2%)
	M1	89 (15.8%)
CEA level, n (%)	≤5	261 (62.9%)
	>5	154 (37.1%)
History of colon polyps, n (%)	No	377 (67.9%)
	Yes	178 (32.1%)
Colon polyps present, n (%)	No	224 (69.3%)
	Yes	99 (30.7%)
Neoplasm type, n (%)	Colon adenocarcinoma	478 (74.2%)
	Rectum adenocarcinoma	166 (25.8%)
Residual tumor, n (%)	R0	468 (91.8%)
	R1	6 (1.2%)
	R2	36 (7.1%)
Lymphatic invasion, n (%)	No	350 (60.1%)
	Yes	232 (39.9%)
Pathologic stage, n (%)	Stage I	111 (17.8%)
	Stage II	238 (38.2%)
	Stage III	184 (29.5%)
	Stage IV	90 (14.4%)
Gender, n (%)	Female	301 (46.7%)
	Male	343 (53.3%)
Primary therapy outcome, n (%)	PD	33 (10.6%)
	SD	5 (1.6%)
	PR	16 (5.1%)
	CR	258 (82.7%)
Age, n (%)	≤65	276 (42.9%)
	>65	368 (57.1%)

**Table 2:** Univariate and multivariate Cox regression analyses of OS in CRC patients

Characteristics	Total (N)	HR (95% CI) Univariate analysis	P value	HR (95% CI) Multivariate analysis	P value
T stage (T3 & T4 vs. T1 & T2)	616	2.406(1.294-4.475)	**0.006**	2.596(0.593-11.362)	0.205
N stage (N1 & N2 vs. N0)	615	2.567(1.787-3.686)	**<0.001**	0.290(0.079-1.068)	0.063
M stage (M1 vs. M0)	545	4.096(2.752-6.096)	**<0.001**	1.797(0.697-4.635)	0.225
Pathologic stage (Stage III & Stage IV vs. Stage I & Stage II)	598	2.916(1.991-4.272)	**<0.001**	7.117(1.543-32.830)	**0.012**
CEA level (>5 vs. ≤5)	396	2.697(1.646-4.418)	**<0.001**	1.657(0.825-3.326)	0.156
Neoplasm type (Rectum adenocarcinoma vs. Colon adenocarcinoma)	618	0.742(0.479-1.151)	0.183		
Age (>65 vs. ≤65)	618	2.023(1.371-2.986)	**<0.001**	3.152(1.525-6.513)	**0.002**
Weight (>90 vs. ≤90)	323	0.756(0.412-1.388)	0.367		
Height (≥170 vs. <170)	304	0.773(0.466-1.282)	0.318		
Gender (Male vs. Female)	618	1.056(0.744-1.498)	0.762		
Race (White vs. Asian & Black or African American)	369	0.933(0.541-1.610)	0.803		
History of colon polyps (YES vs. NO)	531	0.832(0.522-1.326)	0.44		
Colon polyps present (YES vs. NO)	298	1.316(0.777-2.229)	0.307		
Residual tumor (R1 & R2 vs. R0)	491	4.466(2.715-7.347)	**<0.001**	1.920(0.834-4.421)	0.125
TP53 status (Mut vs. WT)	526	1.119(0.768-1.632)	0.558		
KRAS status (Mut vs. WT)	526	0.954(0.657-1.385)	0.805		
PIK3CA status (Mut vs. WT)	526	0.892(0.579-1.375)	0.605		
LINC02560 (High vs. Low)	618	1.494(1.049-2.127)	**0.026**	1.962(1.029-3.741)	**0.041**

## Abbreviations

CRC: colorectal cancer
GTEx: Genotype-Tissue Expression
TCGA: the cancer genome atlas
TME: tumor microenvironment
ssGSEA: single sample gene set enrichment analysis
GSEA: Gene set enrichment analysis
OS: overall survival
PFI: progress free interval
CEA: carcinoembryonic antigen
AJCC: American Joint Committee on Cancer
